# Kinetics of Postpartum Mesenteric Artery Structure and Function Relative to Pregnancy and Lactation in Mice

**DOI:** 10.1007/s43032-020-00402-4

**Published:** 2021-01-07

**Authors:** Natalia I. Gokina, Rebecca I. Fairchild, Nicole M. Bishop, Taylor E. Dawson, Kirtika Prakash, Elizabeth A. Bonney

**Affiliations:** 1 Department of Obstetrics, Gynecology and Reproductive Sciences, University of Vermont, Larner College of Medicine, Given Building, 89 Beaumont Avenue, Burlington, VT 05405 USA; 2 Microscopy Imaging Center, University of Vermont, Larner College of Medicine, 149 Beaumont Avenue, Burlington, VT 05405 USA

**Keywords:** Pregnancy, Postpartum, Vasoconstriction, Vasodilation, Nitric oxide, Vascular structure and distensibility, Breastfeeding

## Abstract

Epidemiological evidence suggests that normal pregnancy in women is associated with decreased cardiovascular risk in later life. Clinical studies have provided evidence that alterations in vascular function and structure are detectable long after delivery. To understand these findings, we examined mesenteric artery reactivity at both early (3 days and 2–4 weeks) and late (12 weeks) postpartum (PP) time points in relation to late pregnancy (LP) and lactation. Vessels from virgin controls, LP, PP, and nursing and non-nursing mothers were tested for responses to phenylephrine (PE), high potassium solutions (high K^+^), and acetylcholine (ACh). Passive arterial distensibility, vessel dimensions, and collagen and elastin content were evaluated for the studied groups. We observed that (1) there was a significant inhibition of vascular reactivity to PE in LP, 3 days and 2 weeks PP vessels that returned to pre-pregnancy levels at 4 and 12 weeks PP; (2) inhibition of NO production in PP vessels restored PE-induced constriction to pre-pregnancy levels; (3) vasodilator responses to ACh were similar at all PP periods; (4) LP and early PP was associated with a persistent increase in arterial distensibility that correlates with a PP-induced reduction in wall collagen, and regressed to pre-conception levels at 12 weeks PP; (5) vessels from non-nursing PP mice demonstrated an increased PE reactivity, diminished responses to ACh, and reduced distensibility compared to breastfeeding mice. These studies provide a timeframe for mesenteric artery adaptations that occur during pregnancy and extend to the PP period, but which may be modified by PP events.

**Supplementary Information:**

The online version contains supplementary material available at 10.1007/s43032-020-00402-4.

## Introduction

Normal pregnancy is characterized by a marked maternal cardiovascular adaptation that is critical for fetal survival and development. In the course of pregnancy, maternal cardiac output is increased in association with blood volume expansion [1–3]. Systemic blood pressure and uterine and peripheral vascular resistance are reduced in part due to increased uterine and systemic vascular dilatation and compliance [1–5]. Some of these maternal adaptive changes may persist postpartum for an undefined period.

Healthy pregnancies are generally associated with low long-term maternal cardiovascular risks postpartum (PP) [6, 7]. Observations of increased cardiac output and decreased peripheral vascular resistance have been reported up to 1 year PP in women [8]. In primiparous women, peripheral vascular compliance remains increased and MAP is reduced 1 year after delivery in comparison to the pre-pregnancy state [9]. In contrast, complicated pregnancies such as pre-eclampsia, pre-term birth, gestational diabetes, and high multiparity are associated with increased risks for hypertension, stroke, and diabetes in later life [3, 7, 10–16].

Recent clinical studies demonstrate that PP cardiovascular changes are advantageous for future pregnancies and are important for women’s health [6, 7, 9]. Causes of such beneficial changes in the maternal vasculature in the PP period remain ill-defined and, in fact, maybe a consequence of adaptation induced by pregnancy [14]. However, some cardiovascular changes may be detectable only in the PP period. Outward remodeling of mesenteric resistance arteries has been reported in 7-day PP mice but was not evident in vessels examined during late pregnancy [17].

In addition, clinical observations suggest that lactation in the PP period may be protective against future cardiovascular and metabolic diseases. PP systemic blood pressure is lower in lactating vs. non-lactating women suggesting that breastfeeding produces some beneficial effects on maternal PP vasculature [18, 19].

Previously, we demonstrated an increased distensibility and reduced arterial wall stiffness in the mesenteric vasculature of 4-week PP mice [20]. In this study, we specifically focused on maternal mesenteric vasculature. Mesenteric arcade vessels contribute to the regulation of peripheral vascular resistance [21, 22]. Mesenteric circulation receives around one-third of maternal cardiac output in pregnancy [23]. In women, mesenteric blood flow is increased during pregnancy by 60–75% and remains elevated compared to pre-pregnancy state 1-year postpartum [24]. These data support the role of mesenteric vasculature in the cardiovascular changes during pregnancy and the PP period.

The goal of the current study was to determine whether these PP changes are evident after shorter (3 days, 2 weeks) or longer (12 weeks) PP periods and whether they are modified by nursing. The specific objectives were to (1) characterize constrictor and dilator function as well as structure and distensibility of mesenteric arteries from aged-matched virgin and PP mice; (2) evaluate the role of NO in reduced contractility of arteries in early PP; (3) assess changes in mesenteric artery function and structure in late pregnancy; (4) define collagen and elastin composition in mesenteric vasculature from virgin vs. PP mice; and (5) study the effect of breastfeeding on changes in vascular function and mechanical behavior of mesenteric vasculature at 2 weeks PP.

## Materials and Methods

### Animals and Preparation of Arteries

**Fig. 1:**
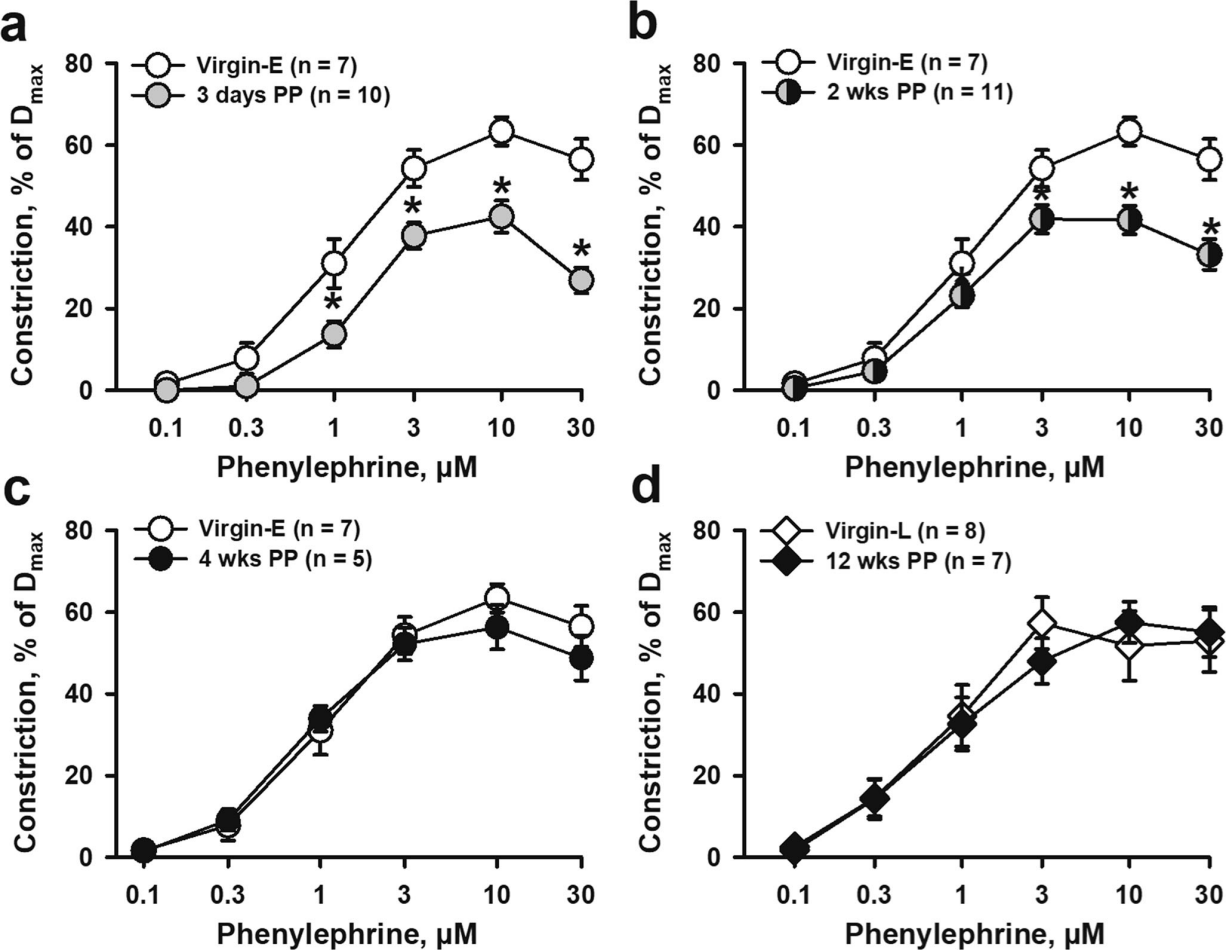
Reactivity of mesenteric arteries to phenylephrine (PE) is reduced in early but not in late postpartum (PP) periods. Summary graphs in **a** and **b** demonstrate a significant reduction in PE-induced sustained vasoconstriction of arteries from 3 days and 2 weeks PP vs. virgin mice. **c** and **d** show the restoration of constrictor responses to PE to pre-pregnancy levels in 4- and 12-week PP mice. PE-induced constriction is expressed as a percentage of initial arterial diameter before PE application. Numbers in parenthesis indicate the number of tested arteries. Virgin-E denotes age-matched virgin controls for the early PP group. Virgin-L states for age-matched virgin controls for late PP. Two-way RM ANOVA analysis was performed for Virgin-E, 3-day, and 2- and 4-week PP groups where the Virgin-E group was accepted as a control. Two-way RM ANOVA analysis was also performed for Virgin-L and 12 weeks PP; the Virgin-L group was used as a control. *Significantly different at *P* < 0.05

All experimental protocols were approved by the Institutional Animal Care and Use Committee of the University of Vermont. C57BL/6 male and female mice were housed at the University of Vermont animal care facility. The source of the mice used for these studies was adult C57BL/6J males and females (Jackson Laboratories #000664). This is a common mouse strain used for genetic studies and is relatively long-lived and is one that we have used previously [25]. For these studies, the descendants of C57BL/6J mice born in-house under specific pathogen-free conditions with regulated light-dark cycles and ad libitum feeding with normal mouse chow (~ 10–14% calories from fat) were aged to 3–5 months, and paired and allowed to mate naturally under the same conditions. Direct use of mice from Jackson laboratory occurred after at least 3 weeks housing in our facility. In some cases, female mice underwent timed mating with males as previously described [26]. After 24 h of mating, females were separated from their male partners (day 0 of gestation) and were used on day 16 of pregnancy or allowed to litter, with the day of littering considered as day one PP. Euthanizing of pups (or fostering to other mothers) within 48 h of birth generated non-nursing mothers. Weaning of pups occurred at 24 days of life. The analysis consisted of vessels from mothers 3 days, 2 weeks, and 4 weeks PP compared with aged-matched controls (Supplement, Fig. 1Sa), consisting of virgin females that were never pregnant and not mated (virgin controls for the early postpartum group, Virgin-E). Similarly, vessels from 12-week PP mice were compared to those from age-matched virgin mice (virgin controls for the late postpartum group, Virgin-L) (Supplement, Fig. 1Sa and 1Sb). Mice at 3 days and 2 weeks PP were heavier than virgin controls. The weights of 4- or 12-week PP females were not significantly different from those of their age-matched virgin controls (Supplement, Fig. 1Sc and 1Sd, respectively).

On the day of experimentation, mice were euthanized by CO_2_ inhalation and cervical dislocation. The mesenteric vascular arcade was quickly removed and placed in pre-aerated (5% O_2_, 10% CO_2_, and 85% N_2_) physiologic salt solution (PSS). Distal segments of second-order mesenteric arteries were removed and cleaned of perivascular fat and connective tissues. Vessels were cannulated from both ends in a pressure arteriograph that was placed on the stage of an inverted microscope with an attached video camera. Arteries were pressurized at 10 mmHg using a Servo pressure system (Living Systems Instrumentation, Burlington, Vermont) and continuously superfused at 3 mL/min with aerated PSS at 37 °C and pH = 7.4 for 1-h equilibration period before starting an experimental protocol.

### Characterization of Mesenteric Vascular Reactivity

Vasoconstrictor and vasodilator arterial responses were studied in all animal groups. After the equilibration period, intraluminal pressure was elevated from 10 to 50 mmHg. To characterize the constrictor function of mesenteric vasculature, we studied arterial diameter changes in response to stimulation of vascular alpha-adrenergic receptors with phenylephrine (PE) in increasing concentrations (0.1–30 μmol/L, 7–10 min for each concentration). Arterial constrictions were also studied in response to gradual depolarization with high potassium (high K^+^) solutions (20–100 mM, 10 min for each concentration). To test vascular endothelial function, acetylcholine (ACh) (0.01–10 μmol/L) was applied in a cumulative fashion (5 min for each concentration) on the arteries pre-constricted with PE to 40–50% of the initial diameter. To clarify the role of nitric oxide (NO) in the modulation of mesenteric vascular function, the responses of arteries from virgin and 2-week PP mice to PE, high K^+^, and ACh were evaluated after 20 min pre-treatment with 200 μM of L-NNA, a nitric oxide synthase (NOS) inhibitor. A combination of diltiazem (10 μmol/L) and papaverine (100 μmol/L) was applied at the end of each experiment to achieve maximal arterial dilatation. Changes in the arterial diameter were continuously monitored using the IonOptix program. Lumen diameters were measured during the last 15–20 s of each tested concentration of drugs using IonOptix software (IonOptix LLC, Westwood, MA). Responses to PE were expressed as the percentage of the initial arterial diameter. In addition, the percentage of maximal PE-induced sustained constriction was used to calculate EC_50_ values. ACh-induced dilatation was expressed as the percentage of maximal dilator response to the application of diltiazem and papaverine or as the percentage of maximal dilator responses to ACh to define EC_50_ values. Data were imported into SigmaPlot program to construct the concentration-response curves for high K^+^-, PE-induced vasoconstriction, and ACh-induced vasodilatation.

### Mechanical Behavior of Arteries

We assessed the passive distensibility of mesenteric arteries from early and late PP mice*.* The arteries were pressurized at 3 mmHg and superfused for 10 min with PSS containing 20 μmol/L diltiazem and 50 μmol/L papaverine to inhibit vascular smooth muscle contractility and to allow for maximal arterial dilation at each pressure tested. The arteries pre-treated with a combination of diltiazem and papaverine did not constrict in response to 100 mM of K^+^ solution. The diameters of arteries from virgin controls (*n* = 6) were 188 ± 5 μm before and 190 ± 6 μm 10 min after high K^+^ application. Similarly, no K^+^-induced constriction was detected in arteries from 2-week PP mice: 227 ± 9 μm before and 230 ± 9 μm after high K^+^ application (*n* = 8).

Inner lumen (*D*_in_) and outer (*D*_out_) arterial diameters were measured after stepwise elevation in intraluminal pressure from 3 to 120 mmHg. Three mmHg is the minimal pressure required to prevent the collapse of an un-stretched vessel. Diameters were measured from images of pressurized arteries on the monitor screen after stabilization of the lumen diameter at each specific level of pressure (typically, 2–3 min for each pressure step). Arterial distensibility was defined as an incremental change in the lumen diameter in response to pressure elevation from 3 to 120 mmHg. The increments in lumen diameter here are expressed as the percentage of an un-stretched vessel diameter at 3 mmHg. We report and compared separately the arterial wall thickness (*t*), calculated as (*D*_out_ − *D*_in_)/2, and the passive lumen diameters at 50 mmHg**.**

### Collagen and Elastin Composition of Mesenteric Arteries

**Fig. 2:**
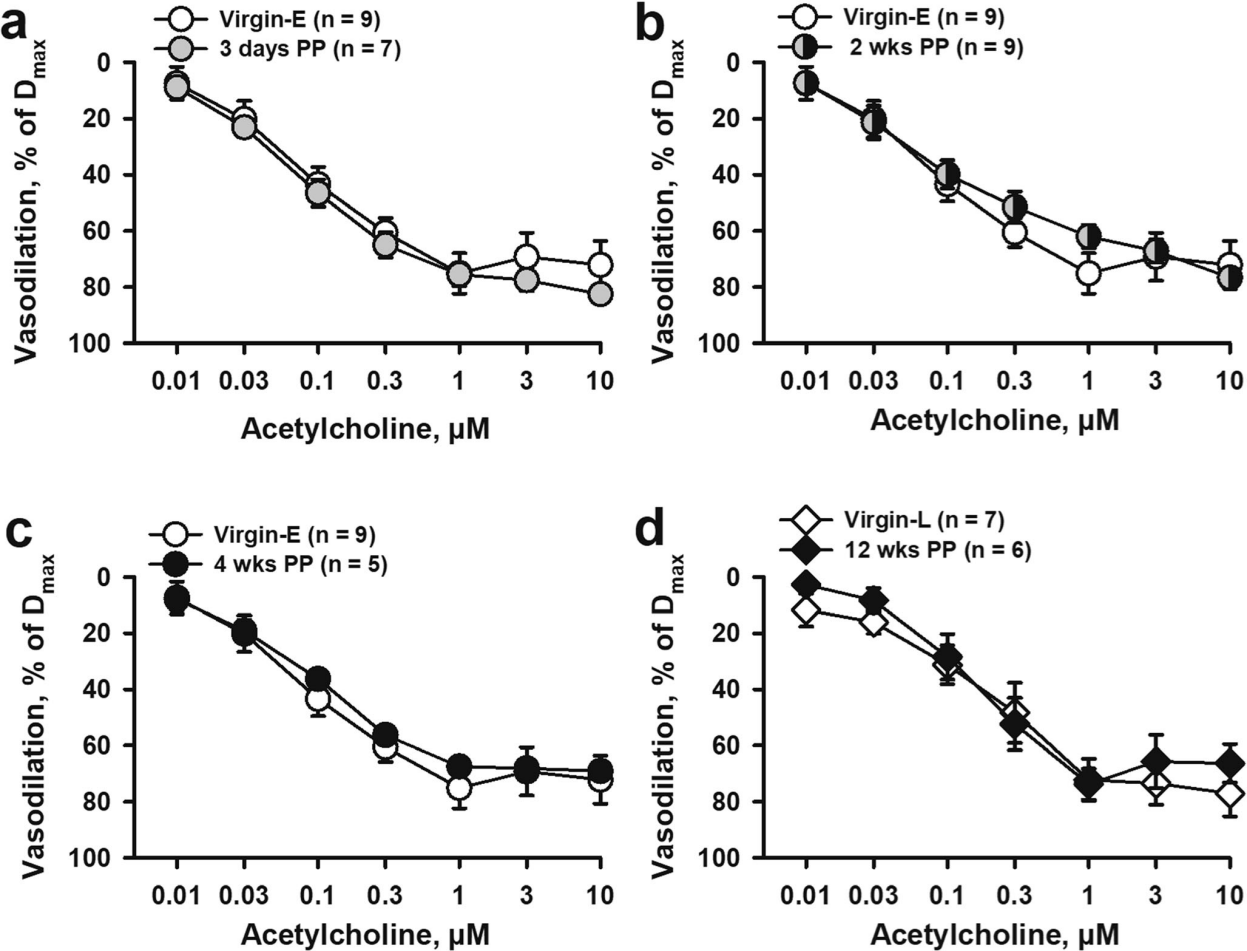
Acetylcholine (ACh)-induced mesenteric artery vasodilation was not affected at early or late PP. Summary graphs showing concentration-dependent vasodilation induced by ACh in mesenteric arteries from 3-day (**a**), 2-week (**b**), 4-week (**c**), and 12-week (**d**) PP mice vs. age-matched virgins. Vasodilation is expressed as a percentage of a maximal dilation of each artery in response to the combined application of papaverine and diltiazem. Numbers in parenthesis indicate the number of tested arteries. Data were compared with two-way RM ANOVA, data from virgin mice were accepted as a control

At the end of each experiment, completely dilated mesenteric arteries at 50 mmHg were fixed in paraformaldehyde for 1 h. Arteries were then embedded in paraffin. Cut sections (3 μm thick) were placed on slides, and de-paraffinized and rehydrated through graded ethanol and distilled water. The slides were then fixed in warmed Bouin’s fixative at 56 °C for 1 h. The slides were stained with Weigert’s iron hematoxylin (to identify nuclei) followed by Verhoeff’s staining solution and 2% ferric chloride (to delineate Elastin). Sodium thiosulfate was used to remove excess iodine before staining with Biebrich scarlet-acid fuchsin (cytoplasm and muscle) followed by phosphomolybdic/ phosphotungstic acid and aniline blue (fibroblast and collagen). After that, slides were washed in running water and treated with 1% acetic acid. Lastly, slides were dehydrated through a series of graded ethanol and xylene before mounting with coverslips using Permount. Tissue sections were visualized using an Olympus BX50 light microscope and images captured with a QImaging Retiga 2000R camera. Images were analyzed using the hue color threshold for both elastin and collagen within MetaMorph (Version 7.10.2.240; Molecular Devices, San Jose, CA) software (Supplement, Fig. 2S).

### Mesenteric Artery eNOS Expression in Virgin and Postpartum Mice

To define the levels of eNOS expression in arteries of virgin and PP mice, the mesenteric vascular arcade was removed and placed in freshly prepared HEPES-PSS (pH 7.4), containing RNA*secure to* irreversibly inactivate RNases (ThermoFisher Scientific, Cat # AM7006). Second-order mesenteric arteries were collected from virgin (*n* = 3) and 2 week PP (*n* = 3) mice. The vessels were cleaned from fat and connective tissues, and residual blood was gently flushed out from the lumen via a small glass cannula. Five to six arteries/per mouse were snap-frozen in liquid nitrogen until processing. The Qiagen RNeasy Micro protocol was followed, including the on column DNAse treatment and eluted in 17 μL warm nuclease-free water. The concentration and integrity of RNA were confirmed by the Promega QuantiFluor RNA System and the Bioanalyzer RNA HS chip. A 1 ng RNA input was used in cDNA synthesis using the ABI High-Capacity cDNA Reverse Transcription Kit. Relative mRNA levels were determined by qRT-PCR using Assays-on-Demand TaqMan Gene Expression Assays (FAM-MGB, ThermoFisher Scientific, Cat # 4331182) for nitric oxide synthase 3 (NOS3 or endothelial NOS, Mm00435217_m1) and β2-macroglobulin (Mm00437762_m1). Values reported are those obtained after normalization to β2-macroglobulin and analyzed by the comparative delta CT method.

### Solutions and Drugs

The physiological salt solution (PSS) contained 119 mmol/L NaCl, 4.7 mmol/L KCl, 24.0 mmol/L NaHCO_3_, 1.2 mmol/L KH_2_PO_4_, 1.6 mmol/L CaCl_2_, 1.2 mmol/L MgSO_4_, 0.023 mmol/L EDTA, and 11.0 mmol/L glucose; pH = 7.4. HEPES-PSS contained 141.8 mmol/L NaCl, 4.7 mmol/L KCl,1.7 mmol/L MgSO_4_, 0.5 mmol/L EDTA, 2.8 mmol/L CaCl_2_, 10.0 mmol/L HEPES, 1.2 mmol/L KH_2_PO_4_, and 5.0 mmol/L glucose; pH was adjusted to 7.4 with NaOH. High K^+^ solutions were prepared by *equimolar* substitution of NaCl with KCl. All chemicals were purchased from Fisher Scientific (Agawam, MA). ACh, PE, diltiazem, and papaverine were obtained from Sigma Chemical Co. (St. Louis, MO). Diltiazem was prepared as a 10 mmol/L stock solution in deionized water and kept refrigerated until use. Stock solutions of L-NNA in PSS and stocks of PE, ACh, and papaverine in deionized water were made on the day of each experiment.

### Statistical Analysis

Statistical analysis of functional and structural data was performed using SigmaPlot version 14 (Systat Software Inc., San Jose, CA). Data are presented as mean ± standard error of the mean. Concentration-response data from different groups of mice were compared by two-way repeated measures ANOVA after passing the Normality Test (Shapiro-Wilk) and Equal Variance Test (Brown-Forsythe). The concentrations of PE and ACh that produced 50% of maximal responses (EC_50_) were defined using SigmaPlot Standard Curve Analysis software (version 14). Summary variables, e.g., lumen diameters, were compared by one-way ANOVA or unpaired *t* test. Hypothesis testing used a probability of *P* < 0.05 as the cutoff for significance.

## Results

### Vasodilator and Vasoconstrictor Reactivity of Vessels from PP Mice

To characterize vascular constrictor and dilator reactivity in early and late PP periods, we tested the arterial responses to alpha-adrenoreceptor agonist phenylephrine (PE) and endothelium-dependent vasodilator acetylcholine (ACh). PE-induced constrictor responses of arteries from virgin, early, and late PP mice are summarized in Fig. 1. Vasoconstrictor reactivity in vessels from 3-day and 2-week PP mice was significantly reduced compared to that of virgin mice (Fig. 1a and b). There was no significant difference in PE-induced constriction detected between virgin and 4-week PP mice (Fig. 1c). PE-induced constriction of mesenteric arteries from late (12 weeks) PP mice was not significantly different compared to that of age-matched virgin mice (Fig. 1d).

To define the effects of PP on the arterial sensitivity to PE, Standard Curve Analysis software was used to calculate EC_50_ values (Fig. 5Sa and 5Sb). No significant difference was found in EC_50_ values for PE-induced constriction of arteries from late gestation and early and late PP mice compared to virgin controls.

Summary graphs shown in Fig. 2 demonstrate that concentration-dependent vasodilation to ACh was not significantly different between virgin and 3-day and 2-, 4- and 12-week PP mice (Fig. 2a–d). EC_50_ values calculated for arteries from virgin controls were also not significantly different from EC_50_ values obtained from vessels at early and late PP (Fig. 5Sd and 5Se).

**Fig. 3:**
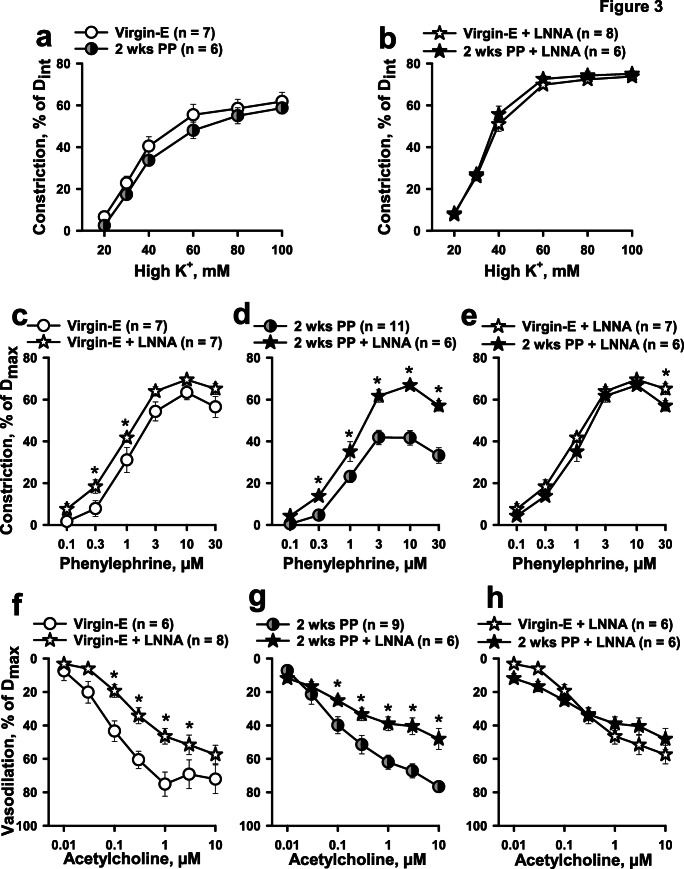
The effects of nitric oxide synthase (NOS) inhibition on constrictor and dilator reactivity of mesenteric arteries from virgin and 2-week PP mice. Concentration-dependent constrictions of mesenteric arteries as a function of increasing concentrations of potassium ions (high K^+^) before (**a**) and after (**b**) inhibition of NO production with 200 μM L-NNA. PE-induced vasoconstriction was significantly enhanced in arteries of virgin (**c**) and PP (**d**) mice pre-treated with L-NNA. Inhibition of NO production restores the PP reactivity to PE to pre-conception levels (**e**). Summary graphs showing a significant attenuation of ACh-induced vasodilation of LNNA-treated vessels from both virgin and PP mice (**f**, **g**). There is no difference in NO-independent dilation to ACh between the pre-conception and the PP periods (**h**). Numbers in parenthesis indicate the number of tested arteries. Data were compared with two-way RM ANOVA; data from virgin mice were accepted as a control. *Significantly different at *P* < 0.05

To determine intrinsic contractility of arteries from virgin and PP mice, the responses to non-receptor activation of VSM contraction with high K^+^ depolarization were studied next. As shown in Fig. 3, there was no significant difference in dose-dependent constrictor responses of vessels from virgin vs. 2-week PP mice (Fig. 3a).

### The Role of NO in Reduced Arterial Contractility During the Early PP Period

To clarify the role of NO in reduced vascular contractility in PP, we tested high K^+^- and PE-induced responses of mesenteric arteries pre-treated with NOS inhibitor L-NNA (200 μM). After blockade of NO production, no difference was detected in responses of arteries from virgin and 2-week PP mice to graded K^+^ depolarization (Fig. 3b). Inhibition of NOS resulted in significant augmentation of PE-induced constriction in vessels from virgin and 2-week PP mice (Fig. 3c and d). L-NNA treatment eliminated the difference in PE-induced constriction between virgin and PP mice (Fig. 3e). NO inhibition also resulted in a significant reduction of vasodilator responses to ACh (Fig. 3f and g). NO-independent dilation to ACh was similar in arteries of both virgin and PP mice (Fig. 3h).

**Fig. 4:**
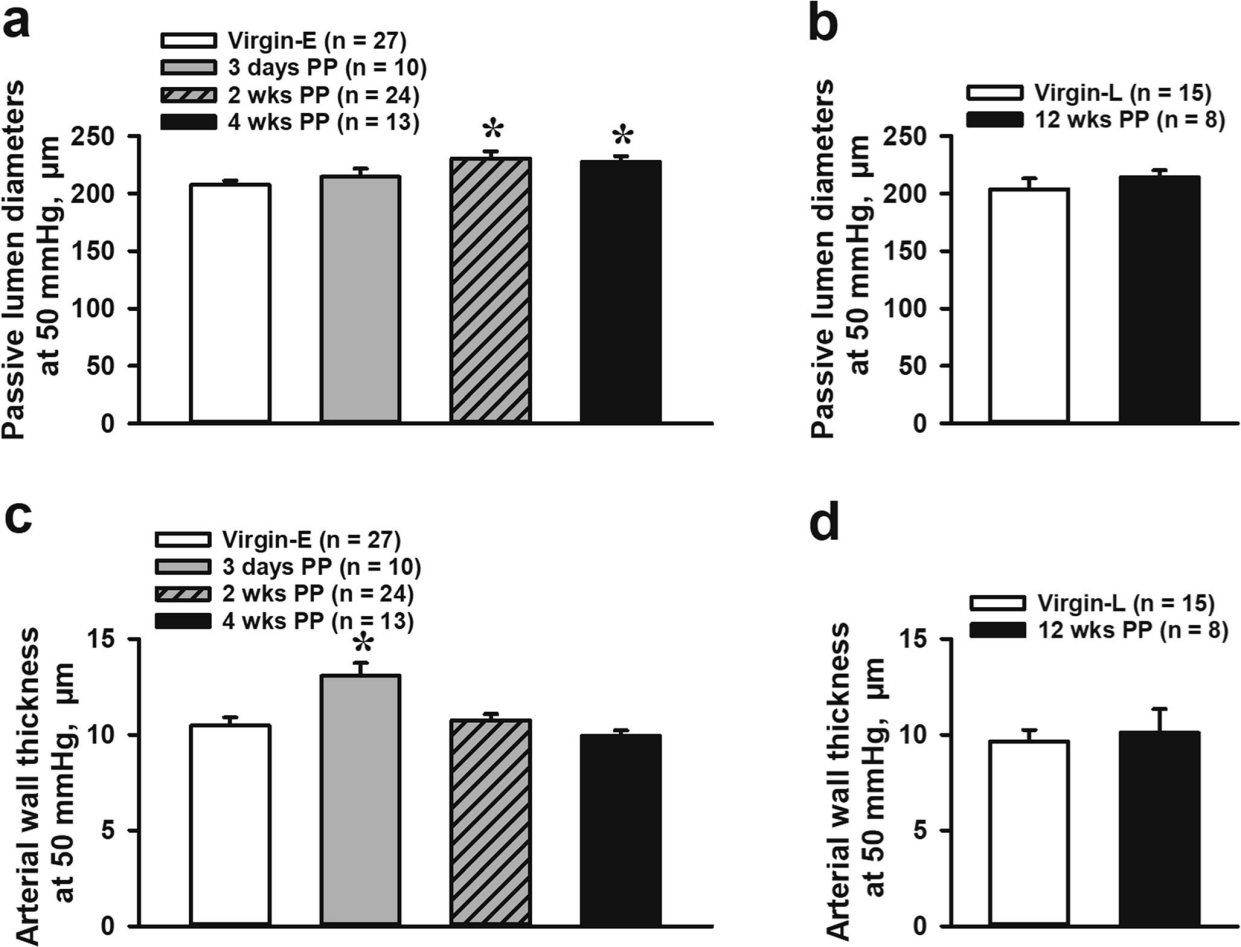
Dynamic changes in passive lumen diameters (**a**, **b**) and wall thickness (**c**, **d**) of mesenteric arteries at different PP periods. All measurements were taken from arteries pressurized at 50 mmHg and relaxed in the PSS containing papaverine and diltiazem. Numbers in parenthesis indicate the number of tested arteries. *Significantly different from virgin controls at *P* < 0.05 (one-way ANOVA or unpaired *t* test)

The role of eNOS expression was evaluated in mesenteric arteries collected from virgin and 2-week PP mice. As shown in Fig. 4S, eNOS expression tend to be higher in vessels from PP compared to virgin mice, although the difference did not reach significance.

### Structural Changes in Mesenteric Vessels from Early and Late PP Periods

Next, we determined structural changes and the mechanical behavior of mesenteric vessels at different PP periods. The passive arterial lumen diameters and arterial wall thicknesses were measured from vessels pressurized at 50 mmHg. There was a trend towards an increase in the passive lumen diameters of mesenteric arteries in the PP period that reached statistical significance at 2 (230.3 ± 6.4 μm) and 4 weeks (227.4 ± 5.0 μm) PP compared to virgin controls (207.5 ± 4.0 μm; Fig. 4a). No difference was detected in passive diameters of arteries from 12-week PP vs. age-matched virgin mice (Fig. 4b). Arterial wall thickness was highest in arteries from 3-day PP mice (13.1 ± 0.7 μm) as compared to vessels from age-matched virgin mice (10.5 ± 0.4 μm) and was gradually reduced to pre-pregnancy levels in 4-week PP mice (9.9 ± 0.5 μm; Fig. 4c). Wall thickness of vessels from 12-week PP mice was not different from that of virgin mice (Fig. 4d).

**Fig. 5:**
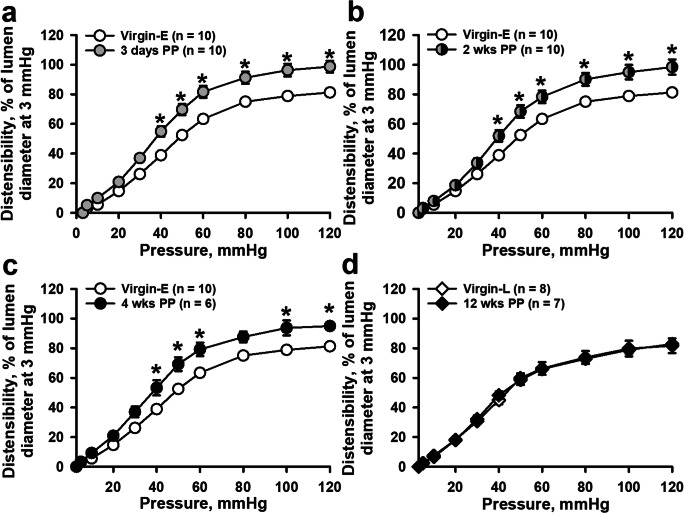
Passive distensibility of mesenteric arteries was significantly increased during early PP (**a**–**c**) and returned to pre-pregnancy levels at late (12 weeks) PP periods (**d**). Passive distensibility is expressed as a percentage of arterial diameters at 3 mmHg (lowest level of pressure that prevents collapsing of the arteries) and plotted as a function of intraluminal pressure. Numbers in parenthesis indicate the number of tested arteries. *Significantly different from virgin controls at *P* < 0.05 (two-way RM ANOVA)

The passive distensibility of mesenteric resistance vasculature was characterized at different PP periods. Figure 5 shows the increments in passive lumen diameters in percentage from lumen diameters of un-stretched vessels (at 3 mmHg) plotted as a function of intraluminal pressure. Distensibility of arteries from 3-day (Fig. 5a), 2-week (Fig. 5b), and 4-week (Fig. 5c) PP mice was significantly increased compared to that of vessels from virgin mice. However, in the late PP period (12 weeks), distensibility of mesenteric arteries was similar to that of vessels from virgin mice (Fig. 5d).

The passive distensibility of arteries is mainly determined by the extracellular matrix components elastin and collagen, which are responsible for extensibility and vessel wall strength, respectively [27]. Therefore, we next turned to a histologic assessment of the collagen and elastin contents in mesenteric arteries from virgin and PP mice.

**Fig. 6:**
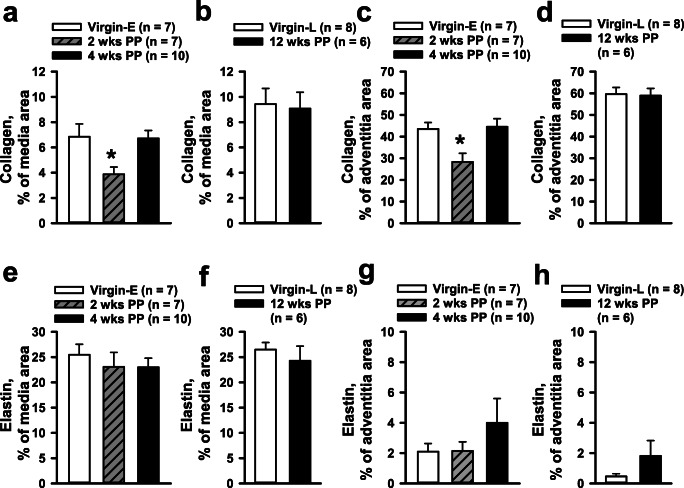
Dynamic changes in collagen and elastin composition in mesenteric artery walls at early and late PP. A significant reduction in the collagen content in the media (**a**) and the adventitia (**c**) at 2 weeks PP was returned to pre-pregnancy levels at 4 and 12 weeks PP (**b**, **d**). No significant changes were detected in elastin content at all PP periods (**e**–**h**). Numbers in parenthesis indicate the number of tested arteries. *Significantly different from virgin (control) group (one-way ANOVA or unpaired *t* test)

We estimated elastin and collagen content as a percentage of the total cross-sectional area of vessel wall media and adventitia. A summary of the contents of collagen and elastin in media and adventitia of mesenteric arteries from different groups of mice is shown in Fig. 6. We observed that collagen is primarily located in the arterial adventitia, as the percentage of this element is almost 10 times higher than that found in the arterial media (Fig. 6a–d). The most dramatic reduction in collagen content was observed in the media and adventitia of vessels from 2-week PP mice. Collagen contents of vessels from 4- and 12-week PP mice, compared to that of vessels from virgins, were not significantly different. We observed that elastin fibers are mainly located in the media of mesenteric arteries with very minor amounts found in their adventitia (Fig. 6e–h). There were no significant differences in the elastin contents of vessels from virgin vs. PP mice.

### Effects of Pregnancy on Function and Structure of Mice Mesenteric Arteries

**Fig. 7:**
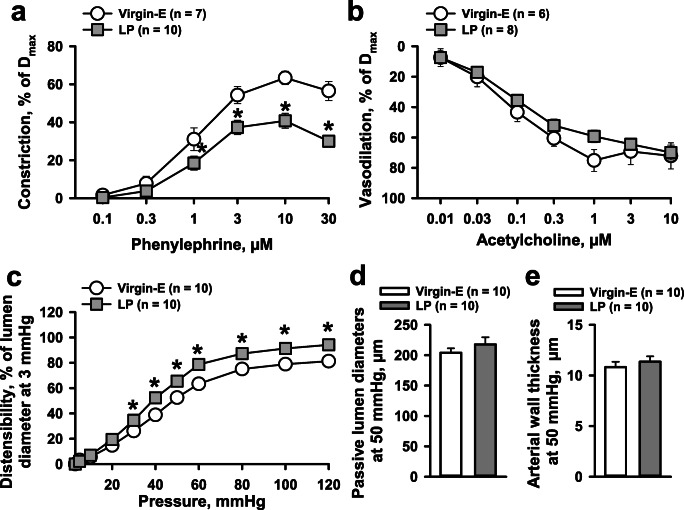
Modulation of function and structure of mesenteric vasculature by late pregnancy in mice. Sensitivity to PE (**a**) was reduced and responsiveness to ACh (**b**) was unchanged in arteries from late pregnant (LP) vs. virgin mice. **c** shows significantly increased passive arterial distensibility in late pregnancy (two-way RM ANOVA). Pregnancy did not affect passive lumen diameters (**d**) and arterial wall thickness (**e**) of mesenteric vasculature (unpaired *t* test). The numbers in parenthesis indicate the number of tested arteries. *Significantly different from virgin controls at P < 0.05

The significant alterations in the PP mesenteric vasculature that we observed may result from changes induced during pregnancy and extended into the PP period [14], or conversely be related to new PP remodeling. Therefore, we also characterized the function and structure of mesenteric arteries from late gestation mice. Summary graphs in Fig. 7 show comparative data from virgin and late pregnant (LP) mice at 16 days of gestation. PE-induced sustained constriction was significantly inhibited in late pregnancy (Fig. 7a). However, we did not detect late pregnancy–related differences in concentration-dependent vasodilation in response to ACh (Fig. 7b). While arterial distensibility was significantly increased in mesenteric arteries from LP as compared to virgin mice (Fig. 7c), no differences were detected in passive lumen diameters or arterial wall thickness (Fig. 7d and e).

### The Effects of Nursing on the Structure and Function of Mesenteric Arteries in the PP Period

Some of our postpartum mice lost their pups soon after delivery (within 48 h, *n* = 5). At 4 weeks PP, vessels from these mice showed distensibility that was similar to that found in virgin vessels and was significantly reduced compared to vessels from nursing mothers (Fig. 3S). These observations suggested that nursing may contribute to changes in function and/or structure of mesenteric arteries during the early PP period. To test this hypothesis, we compared the properties of mesenteric arteries from 2-week nursing vs. non-nursing PP mice whose pups were removed before 48 h PP.

**Fig. 8:**
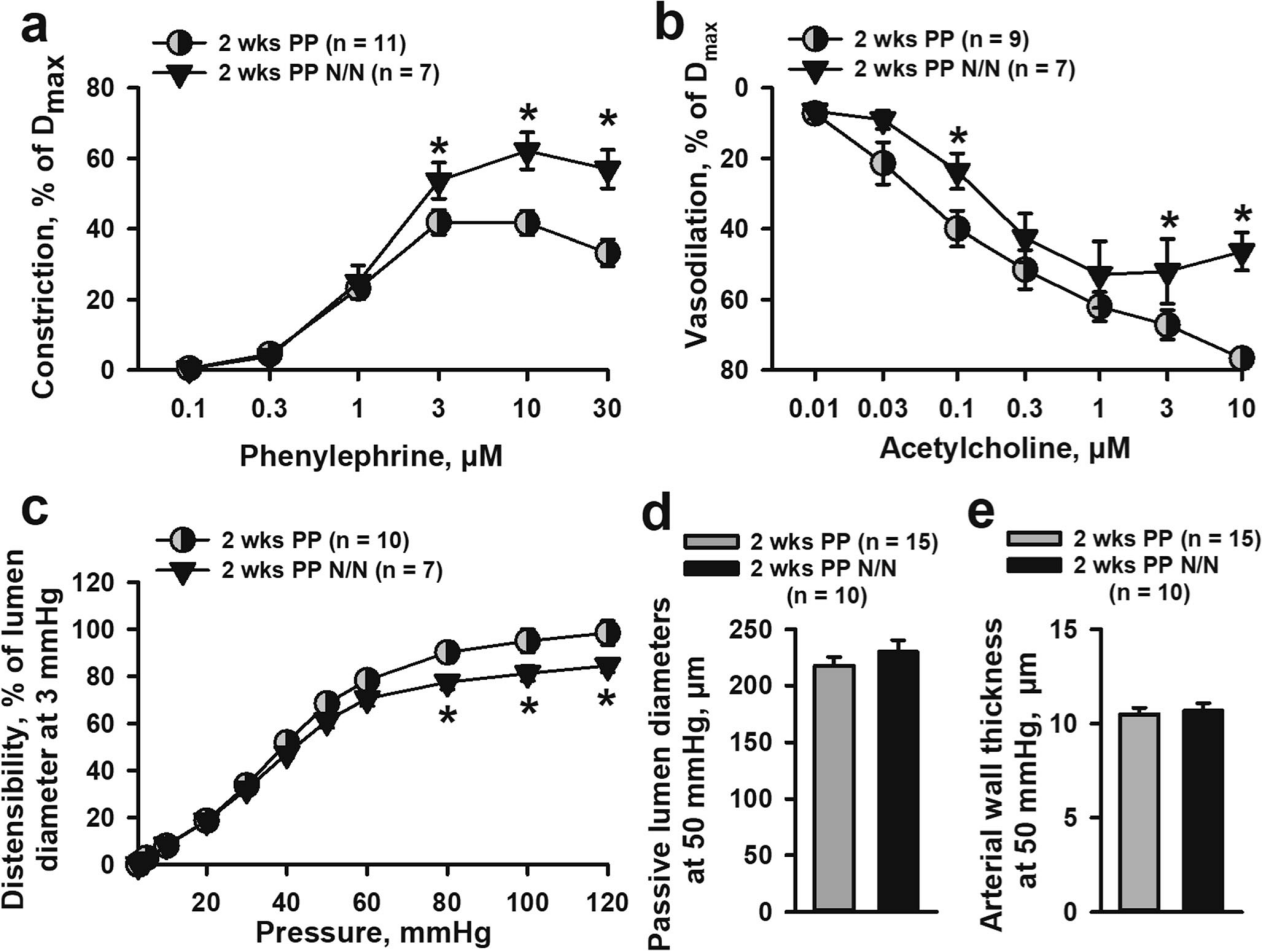
Effects of lactation on function and structure of maternal mesenteric vasculature at 2 weeks PP. Reactivity to PE was significantly increased (**a**) and responses to ACh were reduced (**b**) in arteries from non-nursing (N/N) compared to nursing mice at 2 weeks PP. **c** demonstrates a reduction in passive distensibility of vessels from non-nursing mice (two-way RM ANOVA). Passive lumen diameters (**d**) and wall thicknesses (**e**) were not affected by breastfeeding (unpaired *t* test). The numbers in parenthesis indicate the numbers of the tested arteries. *Significantly different from nursing group at P < 0.05

Figure 8 demonstrates functional and structural changes in arteries from 2-week PP non-nursing mice compared with data obtained from 2-week nursing PP mice. While PE-induced constriction was significantly reduced in vessels from 2-week PP nursing mice as compared to vessels from virgins (Fig. 1b), PE responses in vessels from 2-week PP non-nursing mice were significantly enhanced (Fig. 8a). In contrast to our data from PP mice where we did not detect changes in dilation to ACh, the arteries from non-nursing mice were significantly less sensitive to ACh than vessels from 2 weeks PP (Fig. 8b). The distensibility of these vessels was significantly reduced compared to that of arteries from nursing mothers (Fig. 8c). However, at 2 weeks PP, vessels from non-nursing mothers did not exhibit changes in lumen diameters (Fig. 8d) or arterial wall thickness (Fig. 8e) as compared to vessels from nursing mice.

**Fig. 9:**
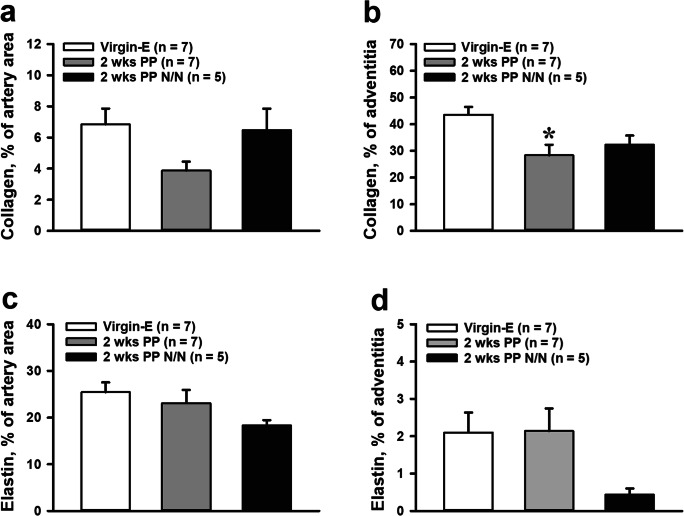
Effects of lactation on collagen and elastin composition of mesenteric arteries from 2-week PP mice. **a** and **b** demonstrate a significant reduction in collagen contents in adventitia of arteries from nursing but not from non-nursing (N/N) mothers. Elastin content trends to reduce in the mesenteric vasculature of non-nursing mothers (**c**, **d**). The numbers in parenthesis indicate the numbers of the tested arteries. *Significantly different from virgin controls at P<0.05 (one-way ANOVA)

Although collagen levels were reduced in vessels from nursing as compared to virgin mice, these changes were absent in vessels of non-nursing mice (Fig. 9a and b). The levels of elastin were lower in both media and adventitia of arteries from non-nursing as compared to vessels from nursing mice; this difference did not reach significance (Fig. 9c and d).

## Discussion

The key findings of this study are the following: (1) early (3 days and 2 weeks) PP period is associated with a significant inhibition of maternal mesenteric artery reactivity to PE that returned to pre-pregnancy levels at 4 and 12 weeks PP. (2) Inhibition of NO production resulted in the restoration of postpartum PE reactivity to pre-conception levels. (3) Vasodilator reactivity of maternal mesenteric arteries to ACh was unaltered at any PP period, and was similar to reactivity found in virgin mice. (4) Early PP was associated with a persistent increase in arterial distensibility that regressed to pre-conception levels at 12 weeks PP and correlated with a reduction in collagen composition of arteries at early PP periods. (5) Mesenteric arteries from LP mice show a reduced reactivity to PE and increased passive distensibility compared to virgin controls. (6) Vessels from 2-week non-nursing PP mice demonstrate an increased PE reactivity, diminished responses to ACh, and reduced distensibility compared to 2-week PP breastfeeding mice.

Normal pregnancy is associated with significant functional changes in maternal mesenteric vasculature that include attenuation of constrictor responses to PE and Angiotensin II (AGTII), and enhanced flow-induced endothelium-mediated vasodilation [17, 28–38]. These changes may importantly contribute to increased mesenteric blood flow and reduced peripheral vascular resistance documented in uncomplicated pregnancies in women [8]. Data on structural changes and mechanical behavior of mesenteric arteries in pregnancy is less conclusive. Increase, no change, or even reduction in passive arterial distensibility of mesenteric arteries from late pregnant rats and mice has been reported [39–41]. These discrepant observations may be related to species (e.g., mouse versus rat) or strain differences in the animals used to characterize the mechanics of mesenteric arteries in pregnancy. Such differences may be related to several factors, such as genetics, environment, hormone responsiveness, or immune regulation.

Much less is known about functional and structural changes in the postpartum maternal vasculature of animals and humans. In previous studies in rats, at 4 weeks PP, the reactivity of mesenteric vessels to vasoconstrictor (PE) or vasodilator (methacholine) was similar to those at pre-conception [42]. The findings reported herein and our previous observations [20] agree with these studies.

However, the data from the current study demonstrates a significantly reduced constrictor responsiveness to PE in mesenteric arteries at 3 days and 2 weeks PP (Fig. 1), while reactivity to PE returned to pre-pregnancy levels by 4 and 12 weeks PP. At the same time, the vasoconstriction to graded depolarization with high K^+^ at 2 weeks PP was not different from vascular responses of virgin controls (Fig. 3a). This data indicates that the intrinsic ability of vascular smooth muscle cells (VSMs) to contract (contractility) is not affected during the PP period. Because the EC_50_ values for PE-induced vasoconstriction were not significantly different between studied groups (Fig. 5S), the data suggest that sensitivity of arteries to alpha-adrenoreceptor stimulation is not modified PP. We conclude that reduced downstream efficacy of vasoconstriction in response to stimulation of alpha-adrenoreceptors is a characteristic feature of LP and the early PP period. It has been shown that LP is associated with reduced mesenteric artery reactivity not only to PE but also to angiotensin II, vasopressin, and other vasoconstrictors. In rats, a decreased PE efficacy in late pregnancy returned to NP baseline by 1 week PP [32, 35, 41, 43–46]. Whether the vasoconstriction to different receptor-stimulated agonists is also attenuated in the PP period remains to be explored.

In resistance vasculature, the contraction of VSM is tightly controlled by numerous mediators and mechanisms generated by vascular endothelial cells [47, 48]. During late pregnancy, the basal production of NO and prostacyclin is significantly enhanced in the endothelium of the peripheral vasculature [49–51]. In addition, inhibition of vascular NO or prostacyclin production in late gestation results in significantly augmented systemic blood vessel reactivity to vasoconstrictors [43, 49–51]. In our study, we used ACh-induced vasodilation as an indicator of endothelial function in mesenteric arteries. In the peripheral vasculature, ACh produces its vasodilator effect via activation of muscarinic receptors on endothelial cells that triggers a cascade of intracellular events resulting in elevation of endothelial cell Ca^2+^, production, and release of several relaxing factors [48]. Herein, we report that the vasodilator responses to ACh were not significantly different at both early and late PP periods compared with the preconception state (Fig. 2), indicating that endothelial function was not affected.

Intriguingly, when we tested the role of NO in inhibiting PE-induced constriction in the early PP period, the blockade of NO generation with L-NNA resulted in the restoration of vasoconstriction to PE to pre-pregnancy levels (Fig. 3e). At the same time, we found that eNOS RNA expression in whole mesenteric arteries of 2 weeks PP tended to be higher, but was not significantly different from that found in (Fig. 4S) virgin vessels. It is formally possible that local enzyme activity may be increased and this would need further study to confirm.

In mouse mesenteric arteries, alpha-adrenoreceptors are located exclusively on VSMs as a direct application of PE to endothelial cells produces no calcium response [52]. In arteries, stimulation of alpha-adrenoreceptors results in generation of second messengers: inositol trisphophate (IP3) and diacylglycerol. These molecues are responsible for intracellular Ca^2+^ elevation and Ca^2+^ sensitization, respectively leading to contraction of VSMs (reviewed in [53]). In mesenteric arteries of mice, IP3 can diffuse from cytosol of VSMs to endothelial cells via myoendothelial gap junctions. This event in turn stimulates endothelial channels, NO release, and vasodilation that limits the PE-induced constriction [52, 54].

In contrast to adrenoreceptor stimulation, the major mechanism of VSM contraction in response to high K^+^ depolarization is influx of Ca^2+^ via voltage-gated Ca^2+^ channels with no IP3 generation [53]. This may explain the lack of differences in vasoconstriction to high K^+^ in virgin and PP mice. Based on the published observations, we speculate that IP3-dependent PE-stimulated endothelial NO production might be a specific feedback mechanism that restricts vasoconstriction in the early PP period more efficiently than in pre-conception. The validation of this concept deserves further investigation.

Inhibition of NO production with L-NNA resulted in a marked reduction in ACh-induced vasodilation but residual vasodilator responses were similar in arteries from virgin and PP mice (Fig. 3f–h). Our findings are in agreement with published observations showing no change in flow-mediated vasodilation of women’s brachial artery before pregnancy and 1-year postpartum [9]. In the context of our data, we speculate that PP-induced attenuation in adrenoreceptor-stimulated vasoconstriction may contribute to the increased mesenteric blood flow observed in 1-year PP women [8].

Our study demonstrates that the early PP period in mice is associated with significant remodeling and change in the mechanical behavior of mesenteric arteries. As shown in Fig. 4a, passive lumen diameters are increased in 2- and 4-week PP mice. In addition, arterial distensibility is significantly increased compared with pre-conception levels (Fig. 5). These changes in the structure and mechanical behavior of arteries may contribute to increased arterial compliance observed in vessels form PP women [9] and supports the role of mesenteric vasculature in the long-term beneficial cardiovascular effects of normal pregnancy.

Elastin and collagen are two major components of the arterial extracellular matrix responsible for extensibility and vessel wall strength, respectively [27]. Collagen is a very stiff protein that limits blood vessel distensibility [55]. We found a significant reduction in collagen levels in both media and adventitia that positively correlates with increased distensibility of mesenteric arteries from early PP mice (Fig. 6a and c). Therefore, reduction in collagen content likely is in part responsible for the increased passive distensibility observed in mesenteric arteries at early 2 weeks PP. These changes in arterial collagen are temporal, as no significant alterations were detected at 12 weeks PP as compared to pre-conception (Fig. 6b and d). At the same time, no changes were found in the elastin contents of the mesenteric arterial wall of PP vs. virgin mice (Fig. 6e–h). Although elastin and collagen contents determine blood vessel distensibility and compliance, other molecules and related interactions contribute to elastin or collagen-mediated vascular elasticity [56–58]. For example, a decrease in cross-linking and stability between collagen molecules occurs in mouse cervix cervical tissue in late pregnancy and leads to increased cervical distensibility [59], and we speculate that this mechanism may additionally contribute to the remodeling of vessels at PP. Structural reorganization of elastic fibers may be another mechanism involved in arterial tissue remodeling during pregnancy [27]. Investigation of cross-linking proteins and elastin structure in the mesenteric vascular extracellular matrix in pregnancy or postpartum is an important focus for future studies.

Reduced PE responsiveness in early PP vessels reported herein may be a consequence of pregnancy-induced modulation of vascular function extended into the early PP period or may result from new coincident regulation to return the vascular system to its pre-pregnancy state. The former suggestion is in line with a significant reduction in PE-induced responses of mesenteric arteries from LP vs. virgin mice (Fig. 7a). Moreover, passive distensibility of mesenteric arteries from LP mice was significantly increased compared with that at pre-conception. Our data are in agreement with an idea that reduced sensitivity to PE and increased passive distensibility of mesenteric arteries in early PP is a consequence (at least in part) of changes induced in mesenteric vasculature during pregnancy.

The idea that new coincident regulation of the vasculature may occur PP is supported by our findings. Although we found no significant changes in passive lumen diameters and the wall thickness of arteries from LP vs. virgin mice (Fig. 7d and e), passive diameters were gradually increased early PP and reached significance at 2 and 4 weeks PP (Fig. 4a). Moreover, arterial wall thickness increased and then decreased PP, consistent with our previous observations [20].

There is experimental evidence that augmented blood flow in the mesenteric circulation results in a subsequent increase in the vessel diameters [60, 61]. Such outward remodeling of mesenteric arteries was preceded by significantly elevated mesenteric blood flow in type-2 diabetic mice [62]. Our data together with that from clinical studies [24] support the hypothesis that outward remodeling of mesenteric arteries is mostly induced by PP as a consequence of increased mesenteric blood flow. There are at least two possibilities to explain PP increased blood flow in mesenteric circulation. The first is redistribution of blood volume from uterine to peripheral circulation after the termination of pregnancy. The second possibility is that increased food intake and gut metabolism required to obtain the nutrients needed for breastfeeding drives increased mesenteric blood flow.

The role of breastfeeding in the modulation of function and structure of mesenteric arteries was assessed via comparison of data from 2-week postpartum nursing vs. non-nursing mice. We found that reduced contractility to PE in nursing mice returned to pre-pregnancy levels in non-nursing mice by 2 weeks PP. In addition, vasodilation to ACh was significantly attenuated in non-nursing vs. nursing PP mice. The relationship between lack of nursing and decreased endothelial responsiveness to vasodilators has yet to be explored in this model. High levels of prolactin, an important hormone related to breastfeeding, are interestingly related to poor endothelial function in postmenopausal women [63], and perhaps there is an interaction between sex hormone stimulation and prolactin that preserves endothelial function in pregnancy and the PP period. In contrast, oxytocin may have direct therapeutic benefit, due to reduction in oxidative stress [64].

Finally, an increase in passive distensibility in early PP was not found in arteries from non-nursing mice (Fig. 8). These data all together demonstrate the importance of breastfeeding in supporting the extension of the vascular effects of pregnancy into the early PP period. Mesenteric vasculature contributes to the regulation of peripheral vascular resistance and systemic blood pressure [21, 22]. Therefore, the reduced contractility and increased compliance of these vessels we observed at early PP only in breastfeeding mice may suggest an important beneficial mechanism that contributes to reduced blood pressure in lactating women. Consistent with this, clinical observations present ample evidence that lactating women show significantly reduced levels of systolic and diastolic blood pressure after normal or complicated pregnancies [18, 19, 65].

Our studies provide a timeframe for mesenteric vascular adaptations that occur during pregnancy and extend into the PP period but respond to modification by PP events such as nursing. Functional changes (NO-dependent attenuation of vasoconstriction) appear to have a different arc (e.g., shorter) than structural changes and appear to be modified by PP events. We could speculate then that specific stressors—emotional, physical, and environmental—may lead to different effects whether they occur during pregnancy or PP. Our studies also suggest potential final common mechanisms by which these vascular changes occur. Future studies will focus on the determination of upstream elements feeding into these mechanisms. Particularly important would be those immediately preceding return to pre-pregnancy biology. Further examination of these elements as they interact with other drivers of postpartum physiology will be important for animal models and clinical care.

## Supplementary Information

ESM 1 (PDF 712 kb)
