# Supplementary material for: Kinetics of Postpartum Mesenteric Artery Structure and Function Relative to Pregnancy and Lactation in Mice
Source: Reprod Sci. 2021 Jan 7;28(4):1200–15. doi: 10.1007/s43032-020-00402-4 (PMC7935827; doi:10.1007/s43032-020-00402-4)
Supplement: Supplementary file 1 — (PDF 712 kb) [file 43032_2020_402_MOESM1_ESM.pdf]

Supplemental material:

Kinetics of Postpartum Mesenteric Artery Structure and Function Relative to Pregnancy and Lactation in Mice

Reproductive Sciences

Natalia I Gokina[1], Rebecca I Fairchild[1], Nicole M Bishop[2], Taylor E Dawson[1], Kirtika Prakash[1], Elizabeth A Bonney[1]

[1]Department of Obstetrics, Gynecology and Reproductive Sciences, and [2]Microscopy Imaging Center, University of Vermont, Burlington, Vermont, 05405 USA

Corresponding author:

Natalia I Gokina PhD

Email: Natalia.Gokina@med.uvm.edu

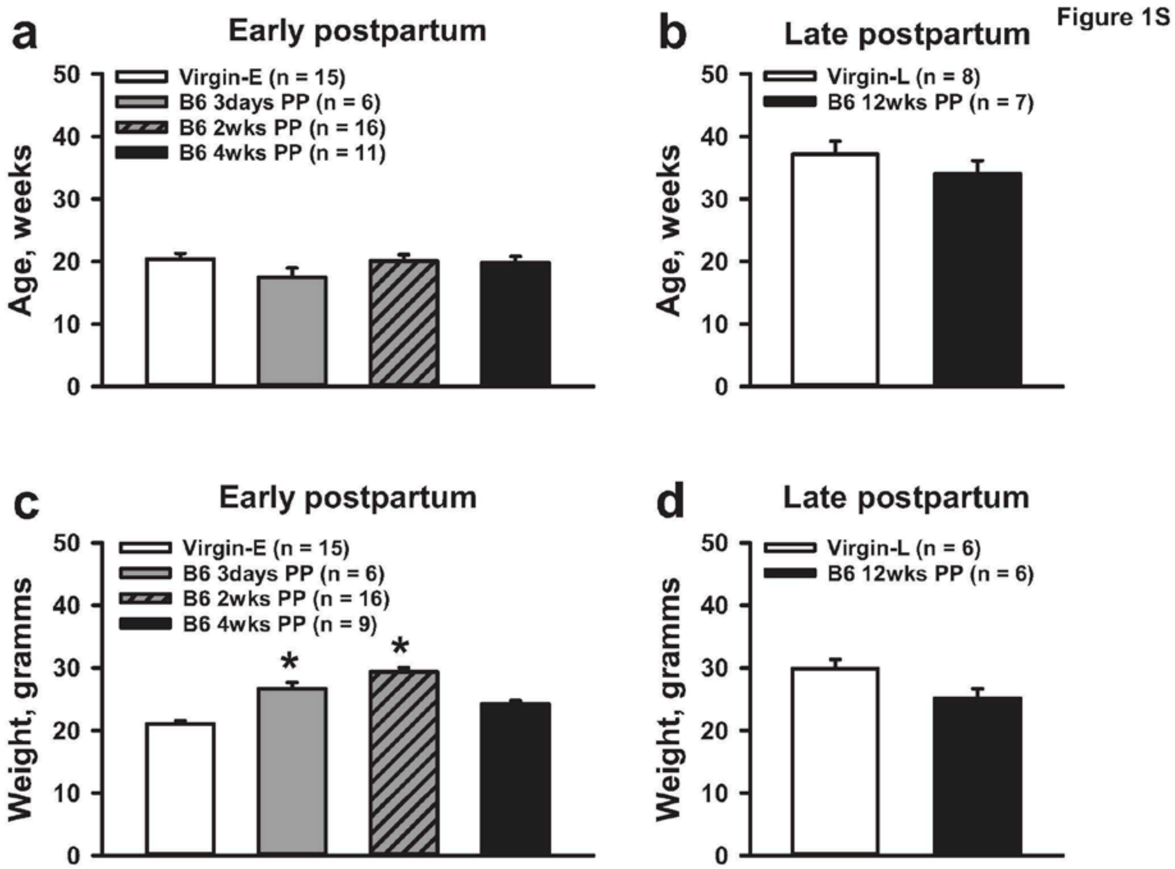

Figure 1S. Characteristics of virgin, early (3-days, 2- and 4-weeks) and late (12-weeks) postpartum (PP) mice. Figures 1Sa and 1Sb show no significant difference in age of mice between virgin and early PP or virgin and late PP groups. The weights were significantly higher in 3-days and 2-weeks nursing PP vs. virgin mice. The weights of mice at 4-weeks PP after weaning pups and cessation of nursing returned to pre-pregnancy levels (Fig. 1Sc). No difference in weights was detected between virgin and 12-weeks PP mice of a similar age (Fig. 1Sd). Virgin-E stands for age-match virgin controls for the early PP group. Virgin-L denotes age-match virgin controls for late 12-weeks PP mice. Numbers in parentheses indicate the number of mice in each studied group.* Significantly different at P<0.05 (One way ANOVA, Figs. 1Sa and 1Sc; t-test, Fig. 1Sb and 1Sd).

Figure 2S

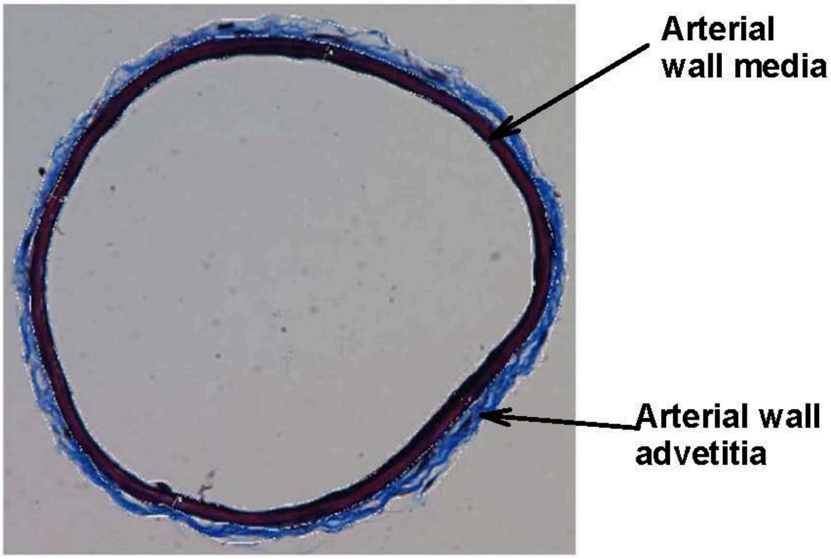

Figure 2S. The representative image of the cross-section of second order mesenteric artery stained using elastic van Gieson and Masson’s trichrome stain. Elastin is stained in purple and collagen is stained in blue. Media and adventitia areas of the artery are depicted by lines in the image.

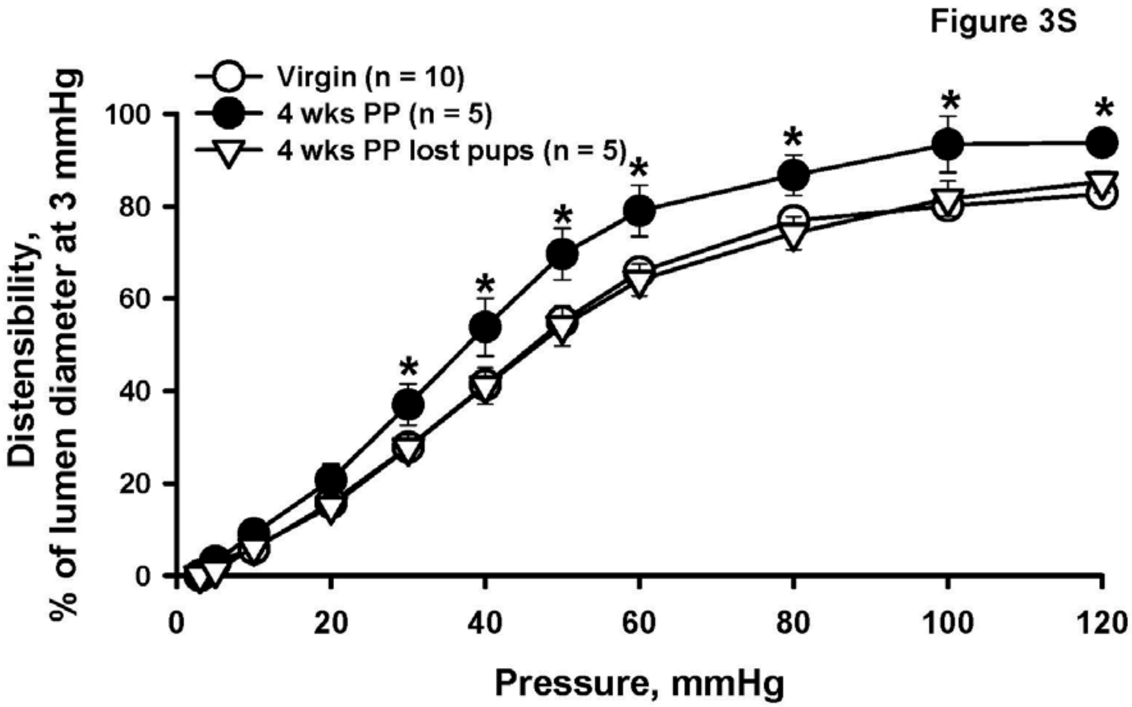

Figure 3S. Passive distensibility of mesenteric arteries from 4-weeks non-nursing mothers approximate the distensibility of vessels from virgin mice. Figure 4S shows passive distensibility of vessels from virgins, 4-weeks PP nursing mothers and 4-weeks PP non-nursing mothers. Numbers in parenthesis indicate the number of tested arteries. Passive distensibility is expressed as a percentage of arterial diameters at 3 mmHg. *Significantly different from virgin controls at $P<0.05$ (two way RM ANOVA).

Figure 4S

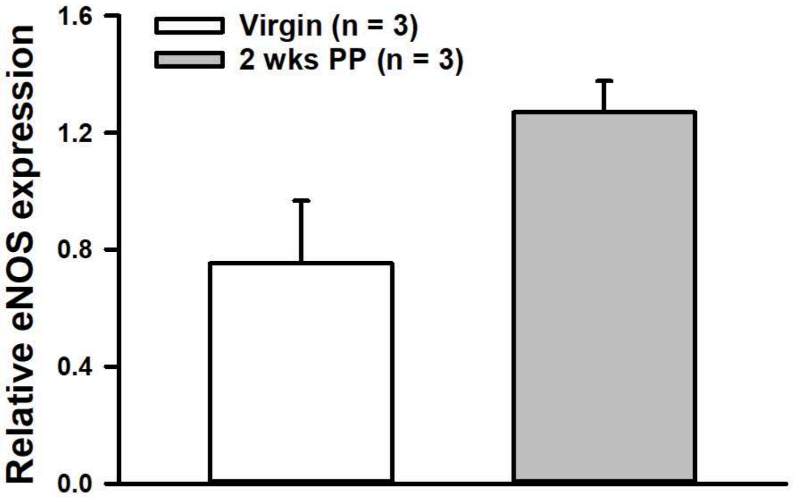

Figure 4S. Relative eNOS expression in mesenteric arteries of virgin and 2-weeks PP mice. Relative mRNA levels were determined by qRT-PCR using Assays–on–Demand TaqMan Gene Expression Assays (FAM-MGB, ThermoFisher Scientific, Cat # 4331182) for nitric oxide synthase 3 (NOS3 or endothelial NOS, Mm00435217_m1) and β2-macroglobulin (Mm00437762_m1. Values reported are those obtained after normalization to β2–macroglobulin and analyzed by the comparative delta CT method.

Figure 5S

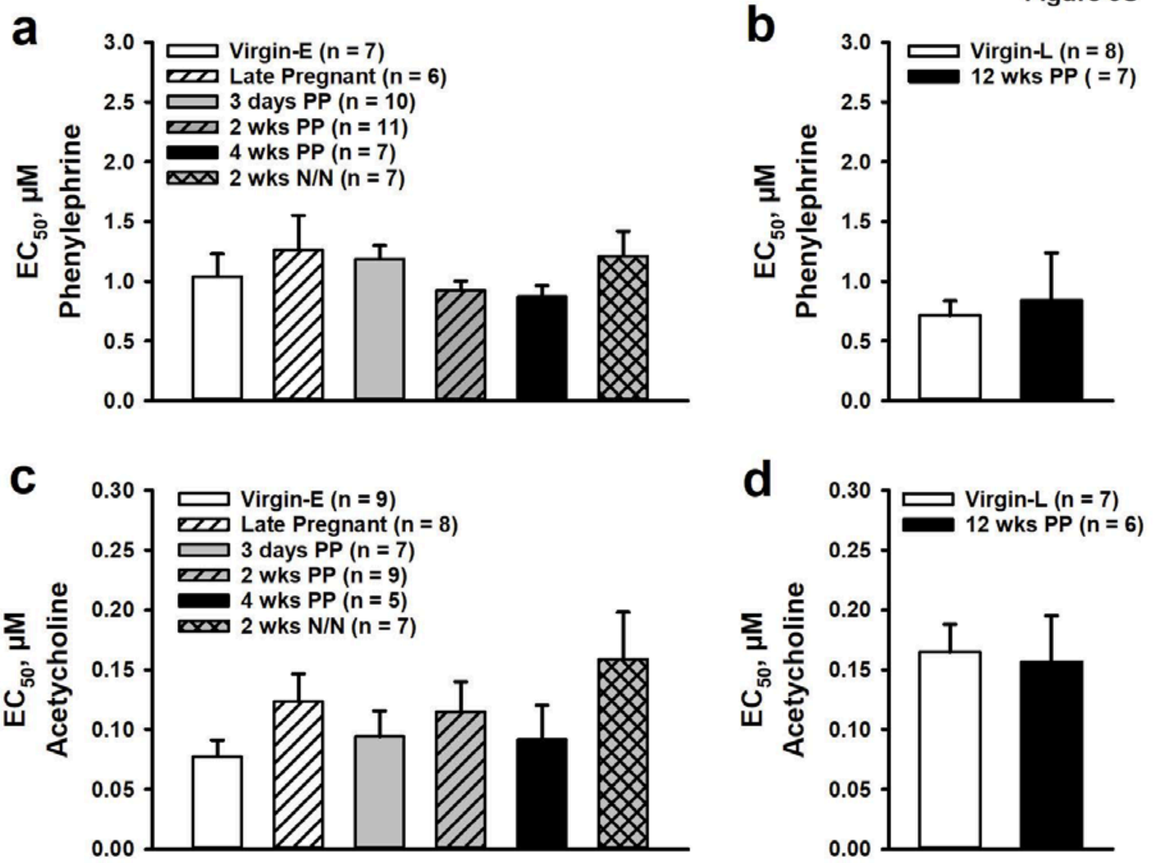

Figure 5S. Concentrations of phenylephrine and ACh that produce 50% effects (EC_{50}) in mesenteric arteries from virgin and postpartum (PP) mice.

EC_{50} values were calculated based on the percentage of agonist-induced concentration-dependent responses in relation to maximal sustained vasoconstriction or vasodilation using SigmaPlot Standard Curve Analysis software (version 14). Virgin-E stands for age-match virgin controls for the early PP group. Virgin-L denotes age-match virgin controls for late 12-weeks PP mice. P values and statistical significance between controls and different PP groups were defined using the Mann-Whitney Rank Sum Test from SigmaPlot analysis program (version 14).
